# Lysosomal and vacuolar sorting: not so different after all!

**DOI:** 10.1042/BST20160050

**Published:** 2016-06-09

**Authors:** Carine de Marcos Lousa, Jurgen Denecke

**Affiliations:** * School of Clinical and Applied Sciences, Faculty of Biomedical Sciences, Leeds Beckett University, Leeds LS13HE, U.K.; † Centre for Plant Sciences, University of Leeds, Leeds LS29JT, U.K.

**Keywords:** endosomal sorting, lysosomes, receptors, trafficking, vacuole

## Abstract

Soluble hydrolases represent the main proteins of lysosomes and vacuoles and are essential to sustain the lytic properties of these organelles typical for the eukaryotic organisms. The sorting of these proteins from ER residents and secreted proteins is controlled by highly specific receptors to avoid mislocalization and subsequent cellular damage. After binding their soluble cargo in the early stage of the secretory pathway, receptors rely on their own sorting signals to reach their target organelles for ligand delivery, and to recycle back for a new round of cargo recognition. Although signals in cargo and receptor molecules have been studied in human, yeast and plant model systems, common denominators and specific examples of diversification have not been systematically explored. This review aims to fill this niche by comparing the structure and the function of lysosomal/vacuolar sorting receptors (VSRs) from these three organisms.

## Discovery and function of lysosomes and vacuoles

The plant vacuole was first discovered in 1676 by a Dutch scientist Antonie van Leeuwenhoek. Considered as the ‘father of microbiology’, he contributed to the development of a number of lenses for microscopes, which allowed him to be the first to observe living cells [1]. Because vacuoles appear as optically empty sacs filling the volume of the cell they have been named from the Latin ‘*vacuus*’ meaning empty. It was only 300 years later that Christian de Duve, a Belgian biochemist, discovered mammalian lysosomes entirely based on biochemical methods [2] and as he calls it himself by ‘a gift of serendipity’ while working on insulin [3]. de Duve named lysosomes respective of their digestive properties (from the Greek *lysis*- digestive and *soma*-body) and their existence was confirmed the following year by electron microscopy [4]. This was the scientific breakthrough that would lead to the understanding of the physiological basis of autophagy and lysosomal storage diseases (LSD) [5,6]. It is now clear that vacuoles in fungi and plants [7,8] have a lot in common with mammalian lysosomes and are highly dynamic in the cell, constantly remodelling with cell cycle stages, growth conditions and cell types [9–13]. There are a few differences that can be noted. Although lysosomes are quite small (0.1–1 um) and numerous (50–1000 per cells depending on the cell type) [14–16] most fungal vacuoles are larger than lysosomes (5 um) and are present in a smaller number (1–5), occupying as much as 20% of the cell volume [10,17]. In contrast, plant vacuoles are large compartments and they can fill 30% and up to 90% of the cell volume [18]. Although it was generally accepted that plant cells have at least two types of vacuoles: a storage vacuole (SV) and a lytic vacuole (LV) [8] this is now being challenged by new findings which favour the one unique vacuole type per cell.

Due to the large size of yeast and plant vacuoles, fluorescence microscopy has become the most widely used tool to study vacuolar trafficking.

Despite a diversity of morphologies and numbers across kingdoms and cell types, lysosomes and vacuoles share a unique and common feature: they represent the essential digestive compartment of the cell. Vacuoles are therefore often referred to as ‘fungal or plant lysosomes’ due to their common role in the degradation of cell components such as proteins, nucleic acids, polysaccharides and lipids. To perform this function, they maintain an acidic pH, between 4.5 and 5.5 for lysosomes and 5.5–6.2 for yeast and plant vacuoles [19–21]. Lysosomes and vacuoles also play roles in protein storage, cell homoeostasis, responses to pathogens, cell signalling, as well as the maintenance of turgor pressure and maintaining general cell shape [22,23]. Typically under conditions of glucose starvation, lysosomes and vacuoles are also involved in autophagy, a mechanism to retrieve energy and building blocks from surplus internal organelles and protein complexes that can be replaced later when glucose is no longer limiting. Whereas vacuoles engulf autophagic vesicles to internalize them, lysosomes are too small to do so and thus fuse with autophagosomes [24].

## Lysosomal and vacuolar lumenal sorting signals

The principal function of lysosomes and vacuole in degradation implies that a number of proteins such as transporters and acid hydrolases have to be efficiently sorted to these compartments. Soluble proteins are diverted from the secretory bulk-flow of proteins due to the presence of sorting signals that are recognized by specific membrane spanning receptors to initiate transport to the degradative organelles. These signals are surface structures either directly displayed by the folded polypeptide (often in fungi and plants) or indirectly via post-translational modification of sugar chains (in vertebrates). Polypeptide signals can vary from 4 to 17 amino acids and although they are often found in an N-terminal propeptide region downstream of the signal peptide for translocation across the ER membrane, C-terminal and even internal domains have been shown to mediate vacuolar sorting.

In the yeast *Saccharomyces cerevisiae*, an N-terminal QRPL motif has been found to target carboxypeptidase (CPY) to the vacuole via the type I membrane spanning receptor Vps10p [25]. Although the glutamate and the leucine seem to be important, the two other amino acids are dispensable, suggesting QXXΦ as consensus signal [26]. However, not all vacuolar proteins in yeast contain such a motif, indicating that alternative signals may exist in yeast [27,28].

Similarly, in plants, a sequence specific vacuolar sorting signal (ssVSS) has been identified in a class of proteins such as barley Aleurain and sweet potato Sporamin, a cysteine protease and a storage protein respectively [29]. It consists of a four amino acids NPIR motif, although some variation may exist. Two additional signals have been described but are still not well understood: a C-terminal vacuolar sorting signal (ctVSS), associated with stretches of hydrophobic residues, and internal targeting sequences forming signal patches [30,31].

In mammals, cathepsin D (aspartyl protease), prosaposin C and acid sphingomyelinase can also interact with their receptor sortilin via stretches of amino acids. In addition, β-glucocerebrosidase (β-GC) is transported to lysosomes by interacting in a similar manner with receptor LIMP-2 [32]. Nevertheless, no consensus motif has been found for these types of proteins.

Mammals, avians, amphibians and invertebrates have developed a non-protein signal [33], based on a modified glycan, the so-called mannose-6-phosphate (M6P) pathway. Lysosomal proteins are translocated into the ER lumen, where the Asparagine in the motif Asn-X-Ser/Thr is glycosylated with an oligosaccharide high in mannose. To distinguish them from secreted glycosylated proteins, M6P formation occurs after arrival in the *cis*-Golgi in two steps: a GlcNac-1-phosphate is transferred from UDP-GlcNac by a phosphotransferase on to selected C6 hydroxyl groups of mannoses generating phosphodiester forms. In the *trans*-Golgi network, the M6P is then exposed by the action of a glucosaminidase: the uncovering enzyme, UCE. The modified glycan is subsequently recognized by M6P receptors (MPRs), large type I membrane spanning proteins that initiate protein export from the Golgi [33,34]. Isosteric analogues of M6P containing two negative charges have also been shown to be a substrate of MPRs [35].

## Lumenal structure and function of lysosomal/vacuolar receptors

**Figure 1:**
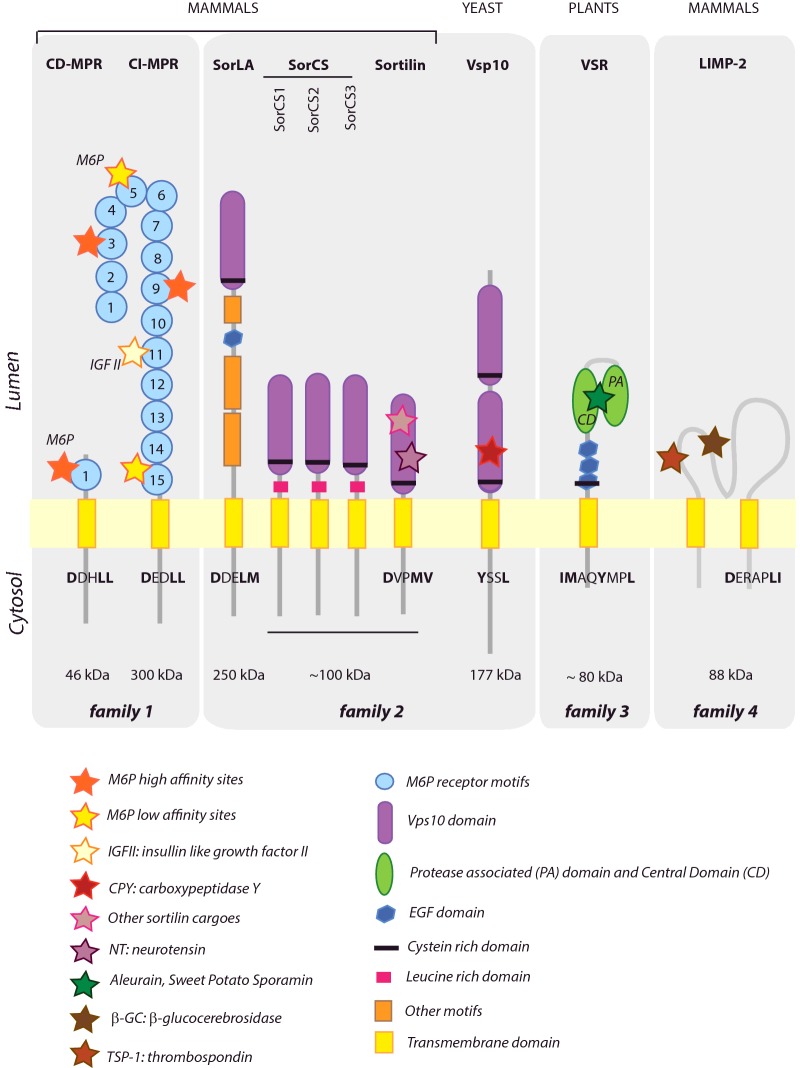
Comparison of lysosomal and vacuolar receptors Mammalian, yeast and plant receptors structures are depicted with structural domain indicated in legend. Sorting signals in C-terminal tails are summarized from [30,34,56] and important amino acids in the motifs are depicted as bold.

Apart from the mammalian receptor LIMP-2, all lysosomal and vacuolar receptors have the same general structure: one large lumenal domain, which is involved in the interaction with the cargo, one transmembrane segment and a C-terminal tail which contains signals for trafficking of the receptor. The N-terminal domain represents the region that is mostly different between receptors among different kingdoms, probably due to the variety of lysosomal/vacuolar proteins to be recognized. Receptors can be classified in four families (Figure 1): i) the MPR family, ii) the sortilin / Vps10 (vacuolar protein sorting 10) family, iii) the plant vacuolar sorting receptor (VSR) family and iv) the LIMP-2 (lysosomal integral membrane protein) family.

Mammalian M6P receptors recognize and bind to a M6P group present on most soluble lysosomal cargoes [36]. A distant MPR related protein (Mrl1p) has been described in yeast but appears to be glycosylation independent [37]. Two receptors have been characterized in mammals: a cation-independent M6P receptor which is approximately 300 kDa (CI-MPR or MPR300) and a cation-dependent M6P receptor which is approximately 46 kDa (CD-MPR or MPR46). CD-MPR contains one domain that can recognize M6P signal, whereas 15 repeats of this one domain are found in the lumenal domain of CI-MPR (Figure 1) [38]. Only two of them however (module 3 and 9) can bind to M6P to similar affinities than the module in CD-MPR [39]. Binding of CD-MPR is enhanced by the presence of divalent ions (hence the name cation-dependent receptor) in contrast with CI-MPR which lacks the critical aspartic acid that coordinates the cation in CD-MPR [38]. Recently, two additional modules (5 and 15) have been found to display weak interaction for M6P and additionally for M6P_GlcNac, the glycosylation intermediate form before M6P. Hence this weak interaction probably represents a rescue mechanisms of lysosomal transport in the event of mutations in the M6P addition process [40,41]. CI-MPR also contains a binding site for insulin growth factor II on module 11, which can inhibit lysosomal cargo binding, suggesting binding site competition. CI-MPR is therefore sometimes called insulin-like growth factor II receptor (IGF II R) [35,42].

The second family, the Sortilin/Vps10 receptor family, has been named after the isolated yeast mutants defective for vacuolar transport (vacuolar protein sorting defective 10). Yeast Vps10p can be considered a specialized form of the much larger and more widespread family called sortilins (Figure 1, family 2). Mammals have five sortilins (sortilin/neurotensin receptor-3, SorC1, SorCS2, SorCS3 and SorLA), but sortilins are also found in most eukaryotic kingdoms including protists, green and red algae, except higher plants. The N-terminal moiety of the yeast Vps10p consists of two domains, currently called Vps10 domains (*Vps10d*). In Vps10p, these can bind differentially to ligands [28]. The sortilins differ mainly in exhibiting just one single *Vps10d*. In mammals, sortilin has been found to bind nerve growth factors and neurotrophin (NT) precursors, and has also recently been involved in the regulation of LDL transport [43]. The four sortilin variants (SorCS1–3 and SorLA) contain other domains among which leucine repeats, EGF repeats, LDLR repeats and β-propeller domain for SorLA, but their presence depends on the type of sortilin and their function is still being examined [44].

The third class of receptors, the plant VSRs [31,42], appears to be separated from the previous mentioned groups and are only found in vascular plants (including mosses and ferns), green unicellular algae, diatoms, watermolds, and the group of protists termed alveolates, but not red algae or other protists. VSRs may have evolved and diversified to compensate for the loss of the sortilin-class receptors in vascular plants. Seven VSRs isoforms exist in *Arabidopsis thaliana*, and they are classified in three groups: VSR1, VSR2 and VSR3 [30]. Genetic studies have shown that the three groups are not functionally equivalent and suggest that VSR3 group might contribute to the transport of a different subset of vacuolar cargoes. The reason for this difference however is not understood as the three families share a high degree of protein identity and structure. The N-terminal region of all VSRs is composed of a PA (protease associated) domain and a central domain (CD), both of which contribute to cargo binding. These are followed by three EGF repeats, which are suggested to be involved in modulating cargo binding through calcium coordination [45]. Finally a single transmembrane connects the lumenal N-terminal part of the protein to a short C-terminal tail of approximately 30 amino acids. The mechanism of cargo binding is still not well understood but it has been shown *in vitro* to depend on the pH and possibility calcium concentrations [46]. A consistently short length of the cytosolic tail has greatly facilitated analysis of the sorting signals that control VSR recycling. A conserved YMPL motif resembles the YXXØ class of clathrin mediated sorting motifs for endocytic recycling from the plasma membrane. However, its role in VSR transport is to prevent arrival at the plasma membrane by directing it from the Golgi apparatus to the multivesicular body and back. Genetic evidence supports a requirement for the tyrosine residue in anterograde trafficking to multivesicular bodies, whereas the hydrophobic leucine is required for retrograde trafficking to earlier compartments [30]. Deletion of the YMPL motif leads to cycling via the plasma membrane to the multivesicular bodies via a conserved IM motif immediately upstream of the YMPL motif that mediates endocytosis [47]. The two sorting signals and their relative positions are extremely conserved among the VSR family (Figure 1, family 3) and the available mutants with distinct sorting properties can now be used to study plant specific aspects of the biosynthetic and endocytic pathways to the vacuole.

The fourth family of lysosomal/vacuolar transporter is uniquely represented by the mammalian model protein LIMP-2 protein (Figure 1, family 4). This is the only lysosomal receptor that is a type III membrane protein with both N- and C-termini in the cytosol and does not appear to have homologues involved in vacuolar trafficking in yeast or plants. LIMP-2 is member of the CD36 scavenger protein family at the cell surface. It is a multicargo receptor: although it can bind to its main cargo, β-GC, through a coiled-coil motif, it can also bind to other cargoes such as TSP-1 and EV71 via a different site localized upstream to the coiled-coil region [48]. Interestingly, LIMP2 lumenal domain is highly glycosylated and a recent study has demonstrated that CI-MPR can interact with the LIMP2–β-GC complex through a specific glycosylation site on LIMP2. This will in turn form a ternary complex which would be the trafficking competent complex [49]. Yet, this hypothesis is in disagreement with earlier reports showing that β-GC trafficking is MPR independent, but could indicate that β-GC could actually use two different trafficking pathways [50].

A striking feature of this four families of receptors is that they all have been suggested to bind their substrates in a pH-dependent manner, where binding would happen at high pH (such as the ER and/or the Golgi) and release would be promoted by acidic pH [late endosomes/multi vesicular bodies (MVBs)] [30,34,38,51,52]. Although the importance of pH in ligand binding/release has been questioned, it is still the preferred model for soluble protein transport to the lysosome and vacuole.

## Lysosomal/vacuolar routes

In contrast with differences in the nature of cargo and the structure of the lumenal domain of receptors, trafficking routes for lysosomal and vacuolar targeting are surprisingly very similar across kingdoms. Besides LIMP-2, which has been reported to bind its substrate in the ER [50], it is generally accepted that most receptors bind lysosomal/vacuolar substrates in the late Golgi compartments, although it is still controversial for the plant VSR [47,53]. After binding to their cargo, the loaded receptors are sorted to the degradative organelles via two routes: the canonical or the alternative route. In the canonical pathway, the receptors are recognized by the adaptor complexes AP1 and/or AP4 at the TGN level. This triggers vesicular targeting to the endosomes in a clathrin dependent pathway [30,34,38,47]. When they reach the late endosomes, the acidic pH triggers the release of the cargo from the receptor, which is then recycled back to the late Golgi by a complex called retromer. In the alternative route, so called ALP pathway in yeast, receptors are recognized by AP3 and reach the lysosome or vacuole by skipping the endosomes, either via the plasma membrane or not [54]. Therefore, receptors using this route are not recycled and only perform one direction delivery [52]. On their way to Lysosomes in either the canonical or alternative pathway, mammalian receptors have been shown to visit the plasma membrane to bind extracellular cargo, and then be endocytosed in an AP2-dependent manner [38]. This is particularly true for multicargo receptors such as CI-MPR, sortilin and LIMP-2 [32,43]. In contrast, yeast Vps10p and plant VSRs do not normally reach the plasma membrane as this will trigger cargo release in the external media, although one plant VSR variant has been found in the plasma membrane in germinating pollen tubes [47,55].

Most receptors using the canonical pathway localize mainly to the endosomes (mostly late Golgi and MVB) with slight variations in ratios, i.e. Vps10p is found in late Golgi whereas VSR is mostly localizing in MVBs [30,56]. Interestingly, CD-MPR and CI-MPR appear to localize to the same region in late Golgi, but different regions in endosomes [57]. Sortilin colocalizes with CI-MPR on endosomes, and both receptors are also partially found in the PM (approximately 10%). This suggests that late endosomes possess specialized areas, one for recycling to the late Golgi (CD-MPR) and one for recycling to the PM (CI-MPR, sortilin). LIMP-2, which uses the alternative pathway and therefore does not reach the MVBs, similarly partially localizes at the PM, but is mostly found in lysosomes [50].

In all trafficking steps, the sorting efficiency depends on the presence of targeting signals in the C-terminal tail of receptors. These signals which will permit interaction with various adaptor protein (AP) complexes. Although minor signals have been reported, two main sorting signals exist across kingdoms: tyrosine signals based YXXΦ motif and dileucine based signals (D/E)XXLL [30,34,38]. Intriguingly in plants, the same YMPL motif seems to mediate anterograde (Golgi to MVBs) and retrograde (MVBs to early trafficking organelles) transport, whereas a second hydrophobic motif (IM: Figure 1) acts as backup mechanism for receptors mistargeted to the plasma membrane. It is not clear if this IM motif relates to the above mentioned dileucine based signals or if it represents a new class of sorting signals [47,58]. In the yeast Vps10p and in lysosomal receptors, anterograde and retrograde sorting appear to be dependent on different motifs [34]. Additionally, the same motifs can be used during endocytosis. The role of adjacent amino acids to the identified sorting signals is now being examined to explain how identical signals can be used to differentially interact with APs involved in different routes. One explanation is the secondary structure that the tail could adopt upon binding to the cargo. This could change depending on the oligomerization state of receptors, as most of these have been shown to dimerize or oligomerize [34]. Moreover, additional signals such as phosphorylations and palmitoylation have been found to play a major role in lysosomal trafficking, but have not been exploited in vacuolar trafficking yet [38,59]. Although cysteines are not present in the C-terminal tail of yeast Vps10p and plant VSRs as potential sites of palmitoylation, they do contain many serines which could be modified by phosphorylation.

## Conclusion

Although lysosomes and vacuoles are morphologically different, they do share a number of crucial functions in human, yeast and plants cells. Their main feature is the lytic degradation of a number of metabolites, which is performed mainly by soluble hydrolases. These proteins are transported to the lysosome and the vacuole by specific receptors. The majority are type I transmembrane proteins composed of a large N-terminal domain, that binds the cargo in early compartments and in a pH sensitive manner, one transmembrane segment and a C-terminal tail, which contains conserved signals for trafficking. Receptors can follow two pathways: a canonical and an alternative pathway. The first route is AP1-dependent and traffics through late endosomes, whereas the latter is AP3-dependent and traffics independent of late endosomes. Hence, despite a large diversification of the lumenal cargo binding domains of the sorting receptors, the three kingdoms appear to be very similar in terms of trafficking cycle, trafficking signals and complexes they interact with along the way (such as AP complexes).

Consequently, yeast and plants are emerging as good alternative organisms for the expression and the purification of human lysosomal proteins with important impacts on pharmaceuticals and enzyme replacement therapy (ERT) [60]. Plants in particular seem to represent a promising system thanks to the cost-effective and easy production of lysosomal proteins with already successful attempts of expressing human hydrolases such as mannosidases and glucocerebrosidase [61,62]. Most importantly, plant-produced (carrot) recombinant human glucocerebrosidase has been shown to naturally possess terminal mannose residues, a step which has to be additionally performed *in vitro* for Chinese hamster ovary (CHO)-derived glucocerebrosidase [63].

Therefore, exploring more interactions between lysosomal and vacuolar fields would provide a step forward into the understanding of trafficking to the lytic organelles and future strategies towards enzyme replacement therapies.

## Abbreviations

β-GC: β-glucocerebrosidase
CHO: Chinese hamster ovary
LIMP-2: lysosomal integral membrane protein
M6P: mannose-6-phosphate
MPR: M6P receptor
MVB: multi vesicular bodies
VSR: vacuolar sorting receptor

## References

[1] van Leeuwenhoek A., Hoole S. (1800). The Select Works of Antony Van Leeuwenhoek, Containing His Microscopical Discoveries in Many of the Works of Nature. Translator;.

[2] Appelmans F., Wattiaux R., de Duve C. (1955). Tissue fractionation studies. 5. The association of acid phosphatase with a special class of cytoplasmic granules in rat liver. Biochem. J..

[3] de Duve C. (2005). The lysosome turns fifty. Nat. Cell Biol..

[4] Novikoff A.B., Beaufay H., de Duve C. (1956). Electron microscopy of lysosome rich fractions from rat liver. J. Biophys. Biochem. Cytol..

[5] Eskelinen E.-L., Saftig P. (2009). Autophagy: a lysosomal degradation pathway with a central role in health and disease. Biochim. Biophys. Acta.

[6] Platt F.M., Boland B., van der Spoel A.C. (2012). Lysosomal storage disorders: the cellular impact of lysosomal dysfunction. J. Cell Biol..

[7] Klionsky D.J., Herman P.K., Emr S.D. (1990). The fungal vacuole: composition, function, and biogenesis. Microbiol. Rev..

[8] Marty F. (1999). Plant vacuoles. Plant Cell.

[9] Zhang C., Hicks G.R., Raikhel N.V. (2014). Plant vacuole morphology and vacuolar trafficking. Front. Plant Sci..

[10] Weisman L.S. (2003). Yeast vacuole inheritance and dynamics. Annu. Rev. Genet..

[11] Weisman L.S. (2006). Organelles on the move: insights from yeast vacuole inheritance. Nat. Rev. Mol. Cell Biol..

[12] Li S.C., Kane P.M. (2009). The yeast lysosome like vacuole: endpoint and crossroads. Biochim. Biophys. Acta.

[13] Bandyopadhyay D., Cyphersmith A., Zapata J.A., Kim Y.J., Payne C.K. (2014). Lysosome transport as a function of lysosome diameter. PLoS One.

[14] Steinman R.M., Brodie S.E., Cohn Z.A. (1976). Membrane flow during pinocytosis: a stereologic analysis. J. Cell Biol..

[15] Steinman R.M., Silver J.M., Cohn Z.A. (1974). Pinocytosis in fibroblasts: quantitative studies *in vitro*. J. Cell Biol..

[16] Bergeland T., Widerberg J., Bakke O., Nordeng T.W. (2001). Mitotic partitioning of endosomes and lysosomes. Curr. Biol..

[17] Hecht K.A., O'Donnell A.F., Brodsky J.L. (2014). The proteolytic landscape of the yeast vacuole. Cell Logist..

[18] Owens T., Poole R.J. (1979). Regulation of cytoplasmic and vacuolar volumes by plant cells in suspension culture. Plant Physiol..

[19] Preston R.A., Murphy R.F., Jones E.W. (1989). Assay of vacuolar pH in yeast and identification of acidification defective mutants. Proc. Natl. Acad. Sci. U.S.A..

[20] Mindell J.A. (2012). Lysosomal acidification mechanisms. Annu. Rev. Physiol..

[21] Martinière A., Bassil E., Jublanc E., Alcon C., Reguera M., Sentenac H., Blumwald E., Paris N. (2013). *In vivo* intracellular pH measurements in tobacco and Arabidopsis reveal an unexpected pH gradient in the endomembrane system. Plant Cell.

[22] Boya P. (2012). Lysosomal function and dysfunction: mechanism and disease. Antioxid. Redox Signal..

[23] Ferguson S.M. (2015). Beyond indigestion: emerging roles for lysosomebased signaling in human disease. Curr. Opin. Cell Biol..

[24] Klionsky D.J., Eskelinen E.L. (2014). The vacuole versus the lysosome: when size matters. Autophagy.

[25] Valls L.A., Winther J.R., Stevens T.H. (1990). Yeast carboxypeptidase Y vacuolar targeting signal is defined by four propeptide amino acids. J. Cell Biol..

[26] van Voorst F., Kielland Brandt M.C., Winther J.R. (1996). Mutational analysis of the vacuolar sorting signal of procarboxypeptidase Y in yeast shows a low requirement for sequence conservation. J. Biol. Chem..

[27] Westphal V., Marcusson E.G., Winther J.R., Emr S.D., van den Hazel H.B. (1996). Multiple pathways for vacuolar sorting of yeast proteinase A. J. Biol. Chem..

[28] Jørgensen M.U., Emr S.D., Winther J.R. (1999). Ligand recognition and domain structure of Vps10p, a vacuolar protein sorting receptor in Saccharomyces cerevisiae. Eur. J. Biochem..

[29] Nakamura K., Matsuoka K. (1993). Protein targeting to the vacuole in plant cells. Plant. Physiol..

[30] De Marcos Lousa C, Gershlick D.C., Denecke J. (2012). Mechanisms and concepts paving the way towards a complete transport cycle of plant vacuolar sorting receptors. Plant Cell.

[31] Pereira C., Pereira S., Pissarra J. (2014). Delivering of proteins to the plant vacuole-an update. Int. J. Mol. Sci..

[32] Saftig P., Klumperman J. (2009). Lysosome biogenesis and lysosomal membrane proteins: trafficking meets function. Nat. Rev. Mol. Cell Biol..

[33] Coutinho M.F., Prata M.J., Alves S. (2012). Mannose 6 phosphate pathway: a review on its role in lysosomal function and dysfunction. Mol. Genet. Metab..

[34] Braulke T., Bonifacino J.S. (2009). Sorting of lysosomal proteins. Biochim. Biophys. Acta.

[35] Gary Bobo M., Nirdé P., Jeanjean A., Morère A., Garcia M. (2007). Mannose 6 phosphate receptor targeting and its applications in human diseases. Curr. Med. Chem..

[36] Kornfeld S., Mellman I. (1989). The biogenesis of lysosomes. Annu. Rev. Cell Biol..

[37] Whyte J.R., Munro S. (2001). A yeast homolog of the mammalian mannose 6 phosphate receptors contributes to the sorting of vacuolar hydrolases. Curr. Biol..

[38] Ghosh P., Dahms N.M., Kornfeld S. (2003). Mannose 6 phosphate receptors: new twists in the tale. Nat. Rev. Mol. Cell Biol..

[39] Tong P.Y., Gregory W., Kornfeld S. (1989). Ligand interactions of the cation independent mannose 6 phosphate receptor: the stoichiometry of mannose 6 phosphate binding. J. Biol. Chem..

[40] Bohnsack R.N., Song X., Olson L.J., Kudo M., Gotschall R.R., Canfield W.M., Cummings R.D., Smith D.F., Dahms N.M. (2009). Cation independent mannose 6 phosphate receptor: a composite of distinct phosphomannosyl binding sites. J. Biol. Chem..

[41] Olson L.J., Castonguay A.C., Lasanajak Y., Peterson F.C., Cummings R.D., Smith D.F., Dahms N.M. (2015). Identification of a fourth mannose 6 phosphate binding site in the cation independent mannose 6 phosphate receptor. Glycobiology.

[42] Morgan D.O., Edman J.C., Standring D.N., Fried V.A., Smith M.C., Roth R.A., Rutter W.J. (1987). Insulin like growth factor II receptor as a multifunctional binding protein. Nature.

[43] Strong A., Patel K., Rader D.J. (2014). Sortilin and lipoprotein metabolism: making sense out of complexity. Curr. Opin. Lipidol..

[44] Hermey G. (2009). The Vps10p domain receptor family. Cell. Mol. Life Sci..

[45] Luo F., Fong Y.H., Zeng Y., Shen J., Jiang L., Wong K.B. (2014). How vacuolar sorting receptor proteins interact with their cargo proteins: crystal structures of apo and cargo bound forms of the protease associated domain from an Arabidopsis vacuolar sorting receptor. Plant Cell..

[46] Watanabe E., Shimada T., Tamura K., Matsushima R., Koumoto Y., Nishimura M., Hara-Nishimura I. (2004). An ER localized form of PV72, a seed specific vacuolar sorting receptor, interferes the transport of an NPIR containing proteinase in Arabidopsis leaves. Plant Cell Physiol..

[47] Gershlick D.C., Lousa Cde M., Foresti O., Lee A.J., Pereira E.A., daSilva L.L.P., Bottanelli F., Denecke J. (2014). Golgi dependent transport of vacuolar sorting receptors is regulated by COPII, AP1, and AP4 protein complexes in tobacco. Plant Cell.

[48] Gonzalez A., Valeiras M., Sidransky E., Tayebi N. (2014). Lysosomal integral membrane protein 2: a new player in lysosome related pathology. Mol. Genet. Metab..

[49] Zhao Y., Ren J., Padilla Parra S., Fry E.E., Stuart D.I. (2014). Lysosome sorting of β glucocerebrosidase by LIMP 2 is targeted by the mannose 6 phosphate receptor. Nat. Commun..

[50] Reczek D., Schwake M., Schröder J., Hughes H., Blanz J., Jin X., Brondyk W., Van Patten S., Edmunds T., Saftig P. (2007). LIMP 2 is a receptor for lysosomal mannose 6phosphate independent targeting of beta glucocerebrosidase. Cell.

[51] Dahms N.M., Olson L.J., Kim J.J.P. (2008). Strategies for carbohydrate recognition by the mannose 6 phosphate receptors. Glycobiology.

[52] Blanz J., Zunke F., Markmann S., Damme M., Braulke T., Saftig P. and, Schwake M. (2015). Mannose 6 phosphate independent lysosomal sorting of LIMP 2. Traffic.

[53] Niemes S., Labs M., Scheuring D., Krueger F., Langhans M., Jesenofsky B., Robinson D.G., Pimpl P. (2010). Sorting of plant vacuolar proteins is initiated in the ER. Plant J..

[54] Rehling P., Darsow T., Katzmann D.J., Emr S.D. (1999). Formation of AP 3 transport intermediates requires Vps41 function. Nat. Cell Biol..

[55] Wang H., Zhuang X.H., Hillmer S., Robinson D.G., Jiang L.W. (2011). Vacuolar sorting receptor (VSR) proteins reach the plasma membrane in germinating pollen tubes. Mol. Plant..

[56] Bowers K., Stevens T.H. (2005). Protein transport from the late Golgi to the vacuole in the yeast Saccharomyces cerevisiae. Biochim. Biophys. Acta.

[57] Klumperman J., Hille A., Veenendaal T., Oorschot V., Stoorvogel W., von Figura K., Geuze H.J. (1993). Differences in the endosomal distributions of the two mannose 6phosphate receptors. J. Cell Biol..

[58] Gershlick D.C., de Marcos Lousa C., Farmer L., Denecke J. (2014). Routes to and from the plasma membrane: bulk flow versus signal mediated endocytosis. Plant Signal. Behav..

[59] McCormick P.J., Dumaresq Doiron K., Pluviose A.S., Pichette V., Tosato G., Lefrancois S. (2008). Palmitoylation controls recycling in lysosomal sorting and trafficking. Traffic.

[60] Miao Y., Ding Y., Sun Q.Y., Xu Z.F., Jiang L. (2008). Plant bioreactors for pharmaceuticals. Biotechnol. Genet. Eng. Rev..

[61] Marchis F., Bellucci M., Pompa A. (2013). Traffic of human αmannosidase in plant cells suggests the presence of a new endoplasmic reticulum to vacuole pathway without involving the Golgi complex. Plant Physiol..

[62] Limkul J., Misaki R., Kato K., Fujiyama K. (2015). The combination of plant translational enhancers and terminator increase the expression of human glucocerebrosidase in Nicotiana benthamiana plants. Plant Sci..

[63] Shaaltiel Y., Bartfeld D., Hashmueli S., Baum G., Brill Almon E., Galili G., Dym O., Boldin-Adamsky S.A., Silman I., Sussman J.L. (2007). Production of glucocerebrosidase with terminal mannose glycans for enzyme replacement therapy of Gaucher's disease using a plant cell system. Plant Biotechnol. J..

